# Study of the Rolling Friction Coefficient between Dissimilar Materials through the Motion of a Conical Pendulum

**DOI:** 10.3390/ma13215032

**Published:** 2020-11-08

**Authors:** Stelian Alaci, Ilie Muscă, Ștefan-Gheorghe Pentiuc

**Affiliations:** 1 Department of Mechanics and Technologies, Stefan cel Mare University of Suceava, 13 University Str., 720229 Suceava, Romania; ilie.musca@usm.ro; 2 Computers Department, Stefan cel Mare University of Suceava, 13 University Str., 720229 Suceava, Romania; pentiuc@usm.ro

**Keywords:** rolling friction coefficient, conical pendulum, damped oscillations, image processing, OpenCV, motion detection

## Abstract

The rolling friction phenomenon is encountered in a wide range of applications and when two different materials are involved, quantitative characterization is necessary. The parameter to be determined is the coefficient of rolling friction, for whose estimation a methodology is proposed, based on the damped oscillation of a conical pendulum. The pure rolling contact between a sphere and a plane is obtained when a steel ball is the bob of the pendulum, which rolls on an inclined plate made from a second material from the contacting pair. The mathematical model of the motion of a conical pendulum constructed from a revolution body supported on an inclined plane in the presence of the rolling friction is developed. The dynamic equations of the rigid body with fixed point are applied and the differential equation of motion of the pendulum is obtained together with the expressions of the reaction forces in the contact point. For different pairs of materials, tests are performed on a laboratory device. The damped oscillatory motion of the conical pendulum is video-captured for the estimation of the angular amplitude variation. A program for image processing is developed for measuring the values of angular elongations from the analysis of each frame of the video and, finally, the coefficient of rolling friction is obtained. For all the materials tested, a linear decrease in angular amplitude is detected and the slope of angular amplitude can be considered as a characteristic parameter related to the coefficient of rolling friction between the two materials.

## 1. Introduction

The elements of any mobile mechanical structure requiring the accomplishment of a definite function must have well expressed motions. To fulfil this condition, the elements of the structure—which are considered rigid bodies as an initial assumption—must come into contact with each other so that, of the six degrees of freedom of the free condition circumstance, some will be cancelled. In the zones of interaction between two elements, according to the action and reaction principle, a system of forces and torques will occur. Their magnitude and orientation are critically influenced by the shape of the contacting surfaces and by the physical properties of the bodies. Adding the condition of smooth surfaces to the hypothesis of rigid bodies, the most general situation is characterized by the theoretical point contact, which is a nonconforming contact. Such contacts are known as higher pairs [1,2] and frequently met in engineering applications. In a higher pair, only the displacement along the common normal in the contact point is not allowed. The possible motions are as follows: sliding along a straight line from the tangent plane and the rotations about the normal (spinning motion), and about a line contained in the tangent plane (rolling motion). To these three motions, the following reactions oppose: the friction force *T* the spinning moment *M_s_* and the rolling moment *M_r_*. The amplitudes of the three components of the friction torsor depend on numerous factors: the magnitude of the normal force, the material properties of the bodies, the quality of the surfaces, the presence of lubrication, etc., [3,4,5]. Of the three components of the torsor, the friction force was the first to be studied. For the dry friction case, the magnitude of the friction force is proportional to the normal force and the friction coefficient is the dynamic sliding coefficient. For the fluid friction case, Newton showed that the amplitude of the friction force is proportional to the velocity gradient [6]. Concerning the magnitude of the spinning torque and rolling torque, in order to explain the occurrence of these, the hypothesis of rigid bodies must be disregarded in favor of the assumption of deformable bodies. A contact surface of small dimensions exists in the vicinity of the initial contact point, and the stress state from the points of this surface can be found based on the theory of elasticity [7,8,9,10]. The two moments can be regarded as the result of elementary moments generated by the contact area’s stress state components. All of the friction torsor’s components consume energy from the mechanical system where they act, and they generate undesirable phenomena such as wear, vibrations, etc. [11,12]. For this reason, knowing the laws describing these parameters as precisely as possible is an essential condition for the correct design of the system so as to ensure optimum running.

Although rolling friction generates less thermal energy and wear compared to sliding friction or spinning friction, its effects are not at all negligible when the contact forces, the angular velocity and the duration of operation of the system have considerable values. The first model for rolling friction is based on the similarity with the dry sliding friction and accepts the proportionality between the rolling friction torque and the normal force from contact [13]. Based on the theory of elasticity, Cherepanov [14] shows that the rolling friction moment is proportional to the normal force raised at power 3/2 for cylinders and at power 4/3 for spheres; the factor of proportionality depends on the elastic characteristics of the materials (Young modulus and Poisson coefficient). Evidently, a complete model should consider all the phenomena influencing the rolling friction: the viscoelastic properties of the two bodies, the presence of adhesion forces, deformations, etc. Quantifying the influence of all phenomena is very difficult; therefore, in engineering applications, a global estimation of rolling friction is expected, accepting that the friction torque depends on the contact loading and on the relative motion from the theoretical point of contact. 

Two main classes of methods can be applied to evaluate the rolling friction torque: the first implies oscillatory motions, and the second uses non oscillatory motions. The first category uses pendula which make contact with the ground [15,16,17,18]. In this case, the estimation of rolling friction moment is based on experimental estimation of the damping of the rotation motion of a pendulum. For the second class, bodies of revolution are used, which roll with angular velocity of constant sign on a support surface, plane or cylindrical. The principle of the method consists in comparing the actual acceleration of the mobile body to the theoretical acceleration, and the difference leads to the estimation of the rolling friction torque [19,20]. Of these two categories of procedures, the oscillatory motion method presents the advantage that the contact zone is reduced; thus, constant parameters of the material and surface are ensured for the entire period of the experiment. 

## 2. Materials and Methods 

### 2.1. Principle of Methodology

**Figure 1:**
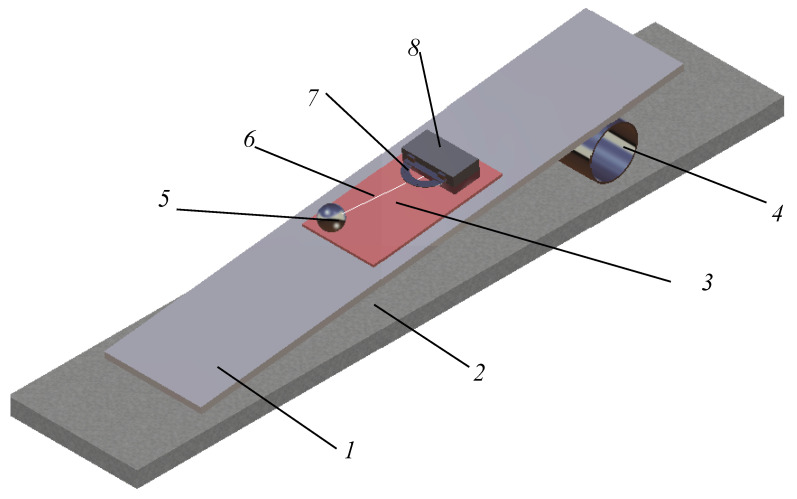
Scheme of the device.

In a moving mechanical system, it was experimentally observed that the attenuation of the motion depends directly on the magnitude of friction from the system. The quantitative characterization of friction effects is one of the tribology objectives and the classical tribometers are based on sliding friction measurements. Referring to the rolling motion, first, the parameters describing the rolling friction phenomenon should be specified, and, afterwards, the methodology for accurate evaluation of these parameters must be detailed. The present work proposes the coefficient of rolling friction as a quantitative parameter influencing the magnitude of the rolling friction torque. For pure rolling motion, the selected moving system consists in a conical pendulum constructed from a ball oscillating on top of a tilted plate; see Figure 1. The rolling friction phenomenon occurs between the spherical bob of the pendulum and the plate. 

**Figure 2:**
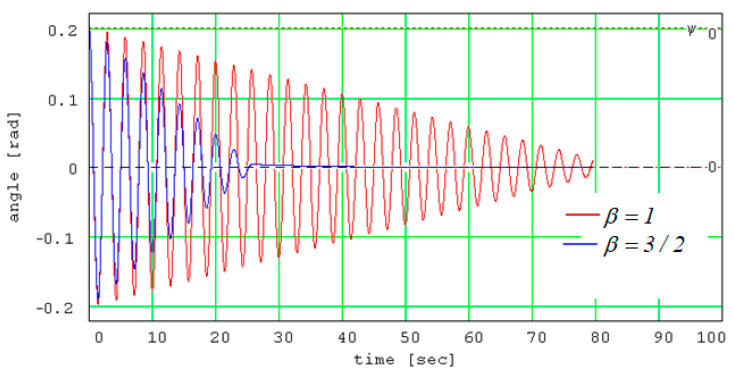
Theoretical solutions for two different dependencies of rolling torque.

The experimental set-up is described in detail in Section 2.3. The aim of the test is to estimate the coefficient of rolling friction between the rolling steel ball and the material to be tested. The experimental damping of the angular amplitudes of the pendulum is corroborated with the theoretical signal obtained by integrating the equation of motion (deduced in Section 2.2) and the value of the coefficient of rolling friction is obtained. The experimental analysis is based on the law of damping of angular amplitude of a pendulum. The theoretical model is developed and the law of motion for a conical pendulum constructed from a sphere linked by a wire to a fulcrum that oscillates on a tilted plane is obtained. The equation of theoretical model permits consideration of any type of dependency between the rolling friction torque and the normal reaction. In Figure 2, there are presented the signals obtained for a power law dependency *M_r_ = s_r_N^β^*, for *β = 1* and *β = 3/2*. For both examples, the angular amplitudes decrease linearly and this remark gives the idea for sketching an experimental set-up for finding the coefficient of rolling friction; see Figure 1. A rectangular thick flat plate, 1, made of duralumin is supported at one end on a horizontal board 2. The other end is positioned at a specified height via a cylinder, 4, placed beneath it. The plate to be tested, 3, is placed on top of the tilted plate, 1. The ball, 5, fixed by an inextensible wire, 6, in a fulcrum attached to the plate, 3 (condition required for pure rolling existence), is brought into contact with the plate, 3. A protractor, 7, fixed with a block, 8, is used for measuring the angular amplitude of the wire. Specifically, for a pendulum that oscillates on a plane tilted with respect to the horizontal, the slope of the straight lines enveloping the damped theoretical angular amplitudes of oscillation must be found and, from here, the value of the coefficient of rolling friction is estimated. 

This paper analyzes, collects and originates results obtained for the coefficient of rolling friction between a steel ball and plates made of different materials (steel, glass, copper, polycarbonate, aluminum, carbon fiber composite) using the methodology described next. 

### 2.2. Proposed Method: Theoretical Fundamentals

**Figure 3:**
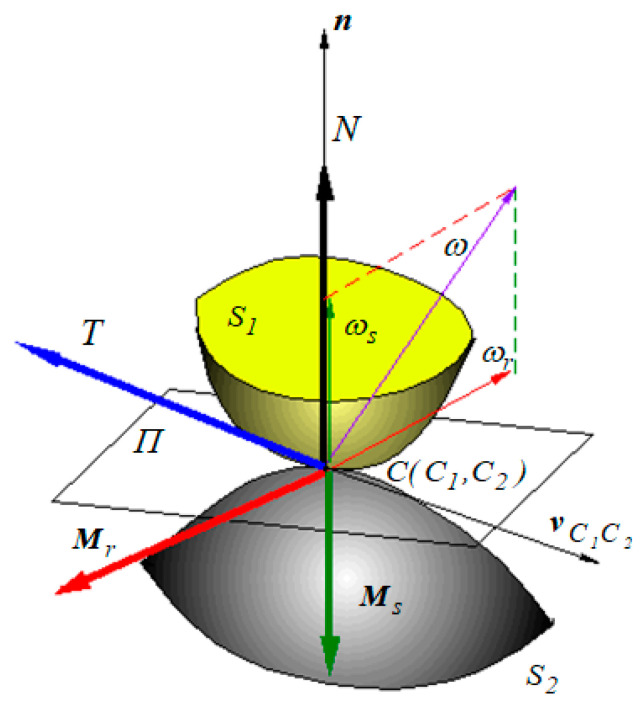
The friction torsor at a point of contact.

Two surfaces *S*_1_, *S*_2_ make non-conformal contact in a point, *C*, where two points, *C*_1_ and *C*_2_, belonging to these, respectively, superimpose; see Figure 3. In the contact point, the tangent plane *Π* is defined and the common normal of versor *n*. The relative motion is characterized by the relative velocity *v*_*C*1*C*2_ between the points *C*_1_, *C*_2_, contained in the tangent plane and by the angular velocity ω which has two components: a component perpendicular to the tangent plane, *ω_s_* (spinning component), and a component contained in the tangent plane, *ω_r_*, the angular rolling velocity. Due to external applied forces, a reaction torsor occurs in the theoretical contact point. In the absence of friction, the interaction between the two surfaces is characterized only by the normal reaction *N* parallel to the normal to the tangent plane. The presence of friction between the two surfaces is described by forces and moments that oppose to the three possibilities of motion:

- to the tendency of sliding between the points *C*_1_ and *C*_2_, the sliding friction force *T*, parallel and of contrary direction of velocity *v*_*C*1*C*2_, will oppose;
- to the spinning motion, the spinning torque *M_s_*, normal to the tangent plane and of contrary direction of the angular velocity *ω_s_*, will oppose;
- to the angular rolling velocity *ω_r_*, the rolling friction torque *M_r_* contained in the tangent plane and of opposite direction of angular velocity *ω_r_*, will oppose.

The components of the reaction torsor at a point of contact are:

- The normal force *N*
- The sliding friction force *T*
- The spinning friction moment *M_s_*
- The rolling friction moment *M_r_*

It was observed that in solving problems from rigid dynamics, the two fundamental theorems (the momentum theorem and the moment of momentum theorem) do not offer sufficient scalar equations for finding all the unknowns of the problem. To surpass this fact, the relations between the components of the friction torsor were demanded. The first one is the unanimously accepted Amonton–Coulomb, which shows proportionality between the modulus of the sliding friction force and the modulus of the normal force:

(1) T=μN.

where *μ* is the dimensionless factor named the coefficient of sliding friction. 

Similar to Equation (1), it was first accepted [13] that the magnitudes of the spinning torque *M_s_* and rolling friction torque *M_r_* can be considered proportional to the modulus of the normal force *N*:

(2) $$M_{s}=s_{s}N,$$

(3) $$M_{r}=s_{r}N$$

where the proportionality factors *s_s_* and *s_r_* have dimensions of length and are known as coefficient of spinning friction and coefficient of rolling friction, respectively. Concerning the rolling friction torques, relatively recent research based on the theory of elasticity showed that *s_r_* from Equation (3) depends on its turn on the normal force ***N***. Regarding the rolling friction moment, *M_r_*, Cherepanov [14] shows that for a spherical rolling body,

(4) $$M_{r}=s_{r}N^{4/3},$$

and

(5) $$M_{r}=s_{r}N^{3/2}$$

for a cylindrical rolling body. Referring to the importance of finding *s_r_*, Cherepanov [14] states, "Experimental determination this coefficient is one of the basic problem of tribology”, and this affirmation justifies the purpose of our work. Recent literature uses a dimensionless coefficient of rolling friction, expressed as *μ_r_ = s_r_/r*, dividing the lever arm coefficient by the value of the radius of the rolling body. 

**Figure 4:**
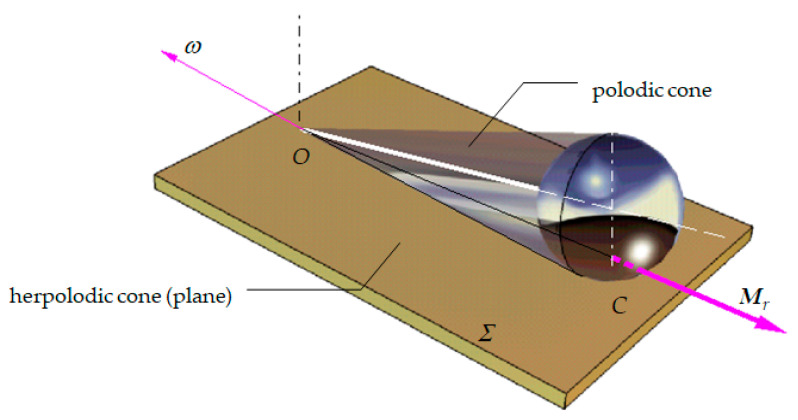
Conical pendulum.

The above considerations are applied for a fixed surface *Σ* and a mobile sphere contacting the surface in the *C* point, as represented in Figure 4. A second point *O* is observed, fixed to the *Σ* surface which, together with the *C* point, defines the instantaneous axis of rotation. The instantaneous axis of rotation defines, with respect to an immobile system and to a system attached to the sphere, the axodes of the motion, which are conical surfaces having *O* as a common point. The relative motion of the two axodes is a pure rolling about the instantaneous axis. For the case of pure rolling, the angular velocity ***ω*** is parallel to the axis of rotation. A consequence of the fact that the angular velocity lies in the plane tangent to the surfaces of the two axodes is the fact that the angular velocity does not have a projection on the normal in the contact point. Therefore, the moment of friction in the contact point is characterized only by the rolling torque *M_r_* collinear to the angular velocity, the spinning torque being zero. When the surface *Σ* is not horizontal and the sphere is tied with an inextensible string in the fulcrum *O*, a conical pendulum is obtained.

**Figure 5:**
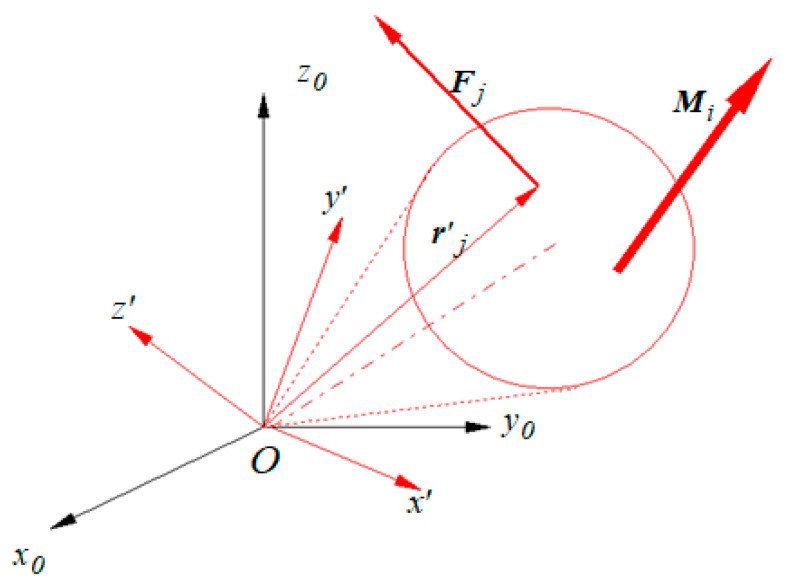
Rigid body subjected to generalized forces.

Assuming a rigid body, as in Figure 5, subjected to *p* external torques *M_i_, i = 1..p* and to *q* external concentrated forces *F_j_, j = 1..q* applied in the points with position vectors *r’_j_, j = 1..q*, the resultant applied moment calculated with respect to *O* is as follows:

(6) $$M_{0}=\sum_{i=1}^{p}M_{p}+\sum_{j=1}^{q}{r^{\prime }}_{j}\times F_{j}.$$

**Figure 6:**
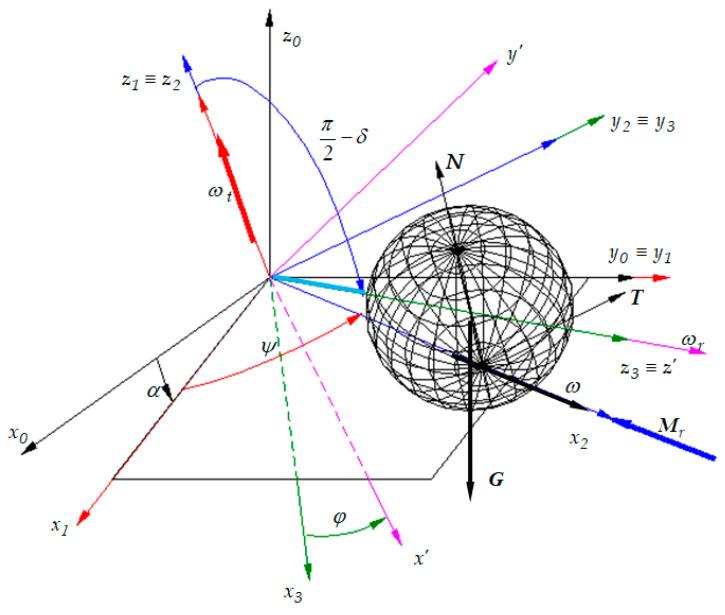
The reference systems used for the model.

For the pendulum bob, the forces are as follows: the weight of the ball *G*, the reaction from the wire *R* (its direction passes through *O* and the moment is zero), the normal reaction *N* and the friction force *T* acting in the point of contact; the moments are the spinning moment *M_s_* parallel to the normal in the contact point (identical zero here) and the rolling friction moment *M_r_* in the tangent plane. The torsion moment from the wire acts along the wire and can be neglected, *M_tw_* ≅ 0 as shown in the Appendix A. The non-zero forces and moments are represented in Figure 6. We examine an inclined plane at the angle α with the vertical direction, as shown in Figure 6. It supports a sphere of *m* mass and *r* radius. An inextensible wire ensures the constant distance *d* between the origin *O* of the system and the center of the ball. The motion of the sphere is modeled by the motion of a rigid with fixed point and described by the moment of momentum theorem [21]:

(7) $$J_{0}\varepsilon +\tilde{\omega }J_{0}\omega =M_{0}.$$

In the above equation, ***J***_0_ is the inertia matrix of the ball with respect to the system *Ox′y′z′* attached to the ball having the axis *Oz′* oriented along the wire; *ω* and *ε* are the column matrices attached to the angular velocity and acceleration, respectively. ῶ is the anti-symmetric matrix attached to the angular velocity vector. Due to the rotational symmetry of the body, the ***J***_0_ matrix is a diagonal one of the following form:

(8) $$J_{O}=\left[\begin{array}{ccc} J_{x} & 0 & 0\\ 0 & J_{y} & 0\\ 0 & 0 & J_{z} \end{array}\right],$$

where

(9) $$J_{z}=\frac{2}{5}mr^{2},$$

and applying the Steiner theorem, the next relation is found:

(10) $$J_{x}=J_{y}=J=J_{z}+md^{2}.$$

The term ***M***_0_ from Equation (7) is a column matrix that has as elements the projections of the resultant moment, on the axes of the mobile frame *Ox′y′z′* attached to the rigid body.

**Figure 7:**
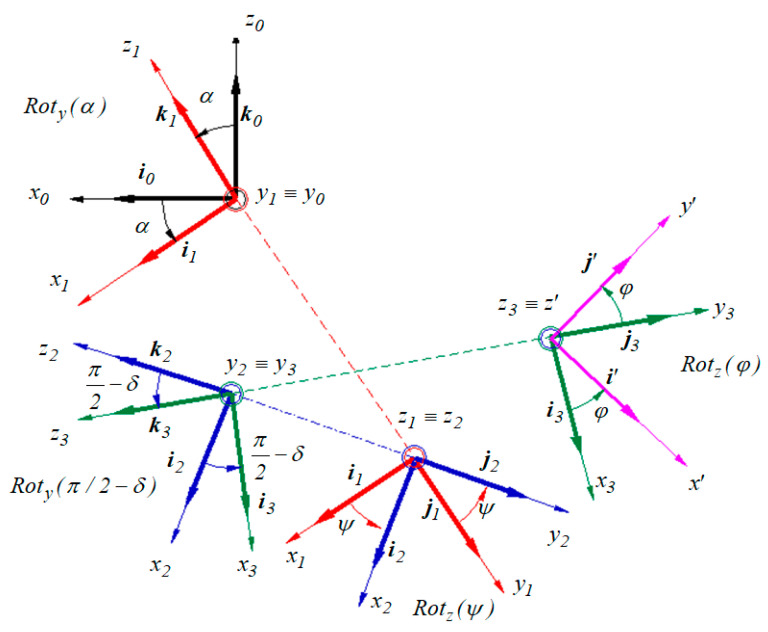
The coordinate systems and the rotations applied to the ball.

Since the scalar equations of motion are needed, the following coordinate systems are considered (Figure 7):

- The *Ox*_0_*y*_0_*z*_0_ system that has the *Oz*_0_ axis in the vertical direction;
- The *Ox*_1_*y*_1_*z*_1_ frame obtained by revolving the frame *Ox*_0_*y*_0_*z*_0_ with an angle *α* about the axis *Oy*_0_ ≡ *Oy*_1_. The ball is sustained by the plane *Ox*_1_*y*_1_;
- The frame *Ox*_2_*y*_2_*z*_2_ obtained by rotating the system *Ox*_1_*y*_1_*z*_1_ around the axis *Oz*_1_ ≡ *Oz*_2_ with an angle *ψ*;
- The coordinate system *Ox*_3_*y*_3_*z*_3_ obtained by rotating the frame *Ox*_2_*y*_2_*z*_2_ with an angle (*π*/2 − *δ*), as shown in Figure 3, about the axis *Oy*_2_ ≡ *Oy*_3_;
- The coordinate system *Ox′y′z′* obtained by rotating the frame *Ox*_3_*y*_3_*z*_3_ with an angle *φ* about the axis *Oz*_3_ ≡ *Oz′*.

The orientation of the axes of the reference system *Ox′y′z′* attached to the rigid with respect to the fix frame is stipulated using three scalar parameters. The system attached to the rigid can be brought into the chosen position if a set of three successive rotations is applied to the rigid body, about three stipulated axes obeying the condition that two successive axes of rotation must not be parallel. The most known sequence is *rot*(*Z*)—>*rot*(*X*)—>*rot*(*Z*) according to Euler’s angles. Another sequence corresponds to Bryant’s angles, *rot*(*X*)—>*rot*(*Y*)—>*rot*(*Z*). Obviously, the use of another rotation combination leads to the same results. In the present work, the rotation sequence was chosen in a manner that, at least in one frame, any of the positional parameters *ψ* and *φ*, kinematical *ω, ε* parameters, constructive parameters *α*, *δ*, *r*, *d*, and the exterior forces and moments *G*, *N*, *T*, *M_r_* must appear in the actual size. The absolute motion of the pendulum could be studied with respect to the fixed reference system *Ox*_1_*y*_1_*z*_1_. However, the use of the fixed coordinate system *Ox*_0_*y*_0_*z*_0_ was necessary in order to express the gravity force *G* in a simple way. In Figure 7, there are presented the four rotations applied to the system *Ox*_0_*y*_0_*z*_0_ in order to bring it to the final position *Ox′y′z′*, passing through the auxiliary (intermediate) systems *Ox*_1_*y*_1_*z*_1_, *Ox*_2_*y*_2_*z*_2_ and *Ox*_3_*y*_3_*z*_3_.

Based on Figure 7, the relations between the versors of the pairs of frames are deduced:

The rotation with an angle *α* about the axis *Oy*_1_ ≡ *Oy*_0_:

(11) $$\left\{ \begin{array}{l} i_{1}=i_{0}\cos\alpha -k_{0}\sin\alpha \\ j_{1}=j_{0}\\ k_{1}=i_{0}\sin\alpha +k_{0}\cos\alpha \end{array}\right.\;\mathrm{or}\;\left\{ \begin{array}{l} i_{0}=i_{1}\cos\alpha +k_{1}\sin\alpha \\ j_{0}=j_{1}\\ k_{0}=-i_{1}\sin\alpha +k_{1}\cos\alpha \end{array}\right.$$

The rotation with an angle *ψ* about the axis *Oz*_1_ ≡ *Oz*_2_:

(12) $$\left\{ \begin{array}{l} i_{2}=\cos\psi \;i_{1}+\sin\psi \;j_{1}\\ j_{2}=-\sin\psi \;i_{1}+\cos\psi \;j_{1}\\ k_{2}=k_{1} \end{array}\right.\;\mathrm{or}\;\left\{ \begin{array}{l} i_{1}=\cos\psi \;i_{2}-\sin\psi \;j_{2}\\ j_{1}=\sin\psi \;i_{1}+\cos\psi \;j_{1}\\ k_{1}=k_{2} \end{array}\right.$$

The rotation with an angle (*π*/2 − *δ*) about the axis *Oy*_2_ ≡ *Oy*_3_:

(13) $$\left\{ \begin{array}{l} i_{3}=\cos(\pi /2-\delta )\;i_{2}-\sin(\pi /2-\delta )\;k_{2}\\ j_{3}=j_{2}\\ k_{3}=\sin(\pi /2-\delta )\;i_{2}+\cos(\pi /2-\delta )k_{2} \end{array}\right.\;\mathrm{or}\;\left\{ \begin{array}{l} i_{2}=\cos(\pi /2-\delta )\;i_{3}+\sin(\pi /2-\delta )\;k_{3}\\ j_{2}=j_{3}\\ k_{2}=-\sin(\pi /2-\delta )\;i_{3}-\cos(\pi /2-\delta )k_{3} \end{array}\right.$$

The rotation with an angle *φ* about the axis *Oz_3_ ≡ Oz’*:

(14) $$\left\{ \begin{array}{l} i^{\prime }=\cos\varphi \;i_{3}+\sin\varphi \;j_{3}\\ j^{\prime }=-\sin\varphi \;i_{3}+\cos\varphi \;j^{\prime }\\ k^{\prime }=k_{3} \end{array}\right.\;\mathrm{or}\;\left\{ \begin{array}{l} i_{3}=\cos\varphi \;i^{\prime }-\sin\varphi \;j^{\prime }\\ j_{3}=\sin\varphi \;i^{\prime }+\cos\varphi \;j^{\prime }\\ k_{3}=k^{\prime } \end{array}\right.$$

The above relations allow for expressing any vector in any of the frames considered and can also be obtained via tensorial calculus [22,23].

**Figure 8:**
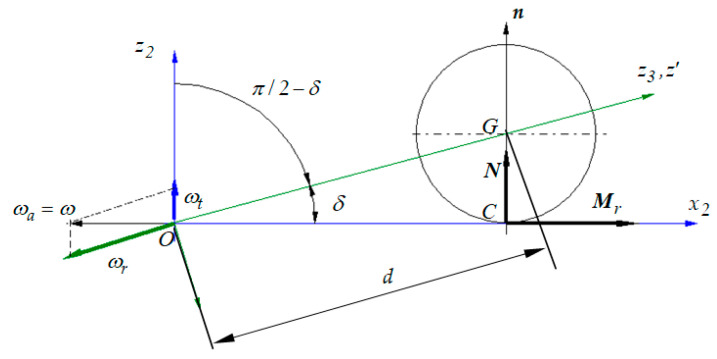
Schematics for the kinematic and dynamic study.

In Figure 8, it can be seen that sin*δ* = *r*/*d* or *δ* = *a*sin(*r*/*d*) and from here, the condition *r*/*d* ≤ 1. The physical significance is obvious: in order to construct the pendulum, the length of the pendulum, measured from the center of the ball to the fulcrum, must be longer than the radius of the ball.

Equations (11)–(14) permit expressing any vector in the frame *Ox′y′z′* attached to the rigid. The angular velocity of the ball can be written as follows:

(15) $$\omega =\dot{\psi }\;k_{1}+\dot{\varphi }\;k_{3},$$

having in the system *Ox′y′z′* the following components:

(16) $$\omega =\left[\begin{array}{l} -\dot{\psi }\cos\varphi \;\cos\delta \\ \dot{\psi }\sin\varphi \;\cos\delta \\ \dot{\psi }\sin\delta +\dot{\varphi } \end{array}\right].$$

The contact point *C* has the following position vector:

(17) $$r_{C}=i_{2}d\cos\delta =\left[\begin{array}{l} \cos\varphi \;\sin\delta \\ -\sin\varphi \;\sin\delta \\ \cos\delta \end{array}\right]d\cos\delta .$$

The pure rolling condition [24,25] in the point *C* is as follows:

(18) $$v_{C}=v_{0}+\omega \times r_{c}=0.$$

Since, in this case, the velocity of the point *O* is zero (the point *O* is immobile), this means that for the pure rolling situation, the position vector of the contact point is parallel to the angular velocity of the ball *ω* with respect to the plane. After the calculus is made, the pure rolling condition in the point *C* is obtained:

(19) $$\dot{\psi }=-\dot{\varphi }\sin\delta .$$

Equations (19) and (16) permit finding the expression of the angular velocity for the pure rolling case:

(20) $$\omega =\left[\begin{array}{c} \dot{\varphi }\cos\varphi \;\sin\delta \\ -\dot{\varphi }\sin\varphi \;\sin\delta \\ \dot{\varphi }\;\cos\delta \end{array}\right]\cos\delta .$$

In order to obtain the vector of angular acceleration, the derivative with respect to time of Equation (20) is made:

(21) $$\varepsilon =\dot{\omega }=\left[\begin{array}{c} \ddot{\varphi }\cos\varphi \;\sin\delta -{\dot{\varphi }}^{2}\sin\varphi \sin\delta \\ -\ddot{\varphi }\sin\varphi \;\sin\delta -{\dot{\varphi }}^{2}\cos\varphi \sin\delta \\ \;\ddot{\varphi }\cos\delta \end{array}\right]\cos\delta .$$

The expression of the left member of Equation (7) can be obtained using Equations (20) and (21):

(22) $$J_{0}\varepsilon +\tilde{\omega }J_{0}\omega =\left[\begin{array}{c} \left[J\ddot{\varphi }\cos\varphi -(J\sin^{2}\delta +J_{z}\cos^{2}\delta ){\dot{\varphi }}^{2}\sin\varphi \right]\cos\delta \sin\delta \\ \left[-J\ddot{\varphi }\;\sin\varphi -(J\sin^{2}\delta +J_{z}\cos^{2}\delta ){\dot{\varphi }}^{2}\cos\varphi \right]\cos\delta \sin\delta \\ \;J_{z}\ddot{\varphi }\cos^{2}\delta \end{array}\right].$$

The right member of Equation (7) must be in explicit form. To this end, the forces and torques acting upon the ball must be identified. The sole external force is ***G***, the weight of the ball,

(23) $$G=-mgk_{0},$$

which acts in the ball’s center of mass, having the vector of position:

(24) $$r_{G}=\;dk_{3}.$$

In the contact point *C* act the following forces:

- The normal reaction ***N***, parallel to ***k***_2_:

(25) $$N=\;Nk_{2};$$

- The friction force ***T*** in the support plane and normal to the angular rolling velocity *ω*:

(26) $$T=\;Tj_{2};$$

- The rolling torque ***M_r_***, collinear to the angular rolling velocity and of opposite orientation:

(27) $$M_{r}=-M_{r}\mathrm{sgn}(\omega )i_{2}.$$

- The spin torque from the contact point is zero since the angular velocity lacks a component along the normal to the support plane.

The reaction of the wire, *R*, passes through the origin of the system of axes and therefore the moment of the reaction with respect to the origin is zero. The unknowns occurring in the system of scalar equations of projection are as follows: one of the angles *φ(t)* or *ψ(t)* (the two angles are related by the pure rolling condition; see Equation (19)), the values of the normal force, the friction force and the rolling friction torque. In order to attain a compatible system of equations, it is assumed that the value of the friction torque is proportional to the value of normal force raised to a certain power.

(28) $$M_{r}=\;s_{r}N^{\beta }.$$

This results in the equation of motion, a nonlinear ordinary differential equation ODE,

(29) $$\ddot{\varphi }=mg\sin\alpha \frac{\sin\psi }{\frac{J_{z}+md^{2}\sin^{2}\delta }{d\sin\delta }\cos\delta }+s_{r}\frac{{\left[\frac{J_{z}+md^{2}\sin^{2}\delta }{d\sin\delta }{\dot{\psi }}^{2}+mg\tan\delta \;\sin\alpha \;\cos\psi \right]}^{\beta }}{(J_{z}+md^{2}\sin^{2}\delta )\cos\delta }\mathrm{sgn}\dot{\psi }.$$

and the reactions from the contact point:

(30) $$N=\frac{J_{z}+md^{2}\sin^{2}\delta }{d}\sin\delta \;{\dot{\varphi }}^{2}+\frac{\sin\alpha \sin\delta \cos\psi +\cos\alpha \cos\delta }{\cos\delta }mg,$$

(31) $$T=\frac{\frac{J_{z}}{md^{2}}}{\frac{J_{z}}{md^{2}}+\sin^{2}\delta }\sin\alpha \sin\psi \;mg-\frac{s_{r}}{d}{\left[\frac{J_{z}+md^{2}\sin^{2}\delta }{d\sin\delta }{\dot{\psi }}^{2}+\tan\delta \sin\alpha \cos\psi \;mg\right]}^{\beta }\frac{\sin\delta \mathrm{sgn}\dot{\psi }}{\frac{J_{z}}{md^{2}}+\sin^{2}\delta }$$

In order to integrate the equation of motion, Equation (29), the derivative of Equation (19) with respect to time, must be considered:

(32) $$\ddot{\psi }=-\ddot{\varphi }\sin\delta .$$

The final form of the equation of motion for pure rolling is as follows:

(33) $$\ddot{\psi }=-mg\sin\alpha \tan\delta \frac{\sin\psi }{\frac{J_{z}+md^{2}\sin^{2}\delta }{d\sin\delta }}-s_{r}\tan\delta \frac{{\left[\frac{J_{z}+md^{2}\sin^{2}\delta }{d\sin\delta }{\dot{\psi }}^{2}+mg\tan\delta \;\sin\alpha \;\cos\psi \right]}^{\beta }}{\left(J_{z}+md^{2}\sin^{2}\delta \right)}\mathrm{sgn}\dot{\psi }.$$

In the solutions (30), (31) and (33) of Equation (7), the second derivative of the position parameter of the system *ψ* occurs only in the last equation. In addition, the expressions of the two reactions *N* and *T* also lack precessional angular acceleration (the second derivative with respect to time of *ψ* angle). This observation permits a substantial simplification of the numerical integration of the equation of motion. When the planar motion pendula are used, the angular acceleration can be found only for the assumption of proportionality between the rolling friction torque and the normal force. Thus, one can conclude that one of the main advantages of the proposed model is that it permits the integration of the equation of motion regardless of the accepted dependency between the rolling friction moment and normal force. Equation (33) is a nonlinear ordinary differential equation (ODE). The Runge–Kutta method [26] is applied in order to integrate it. After the numerical integration, the dependency on time of the precession angle *ψ* and of the precession velocity is found. With these dependencies, the time variations of the friction force and normal reaction are found. These values are required for the validation of pure rolling condition:

(34) $$\frac{\left|T\right|}{N}<\mu ,$$

where *μ* is the coefficient of sliding friction.

### 2.3. Experimental Method

#### 2.3.1. Experimental Device

**Figure 9:**
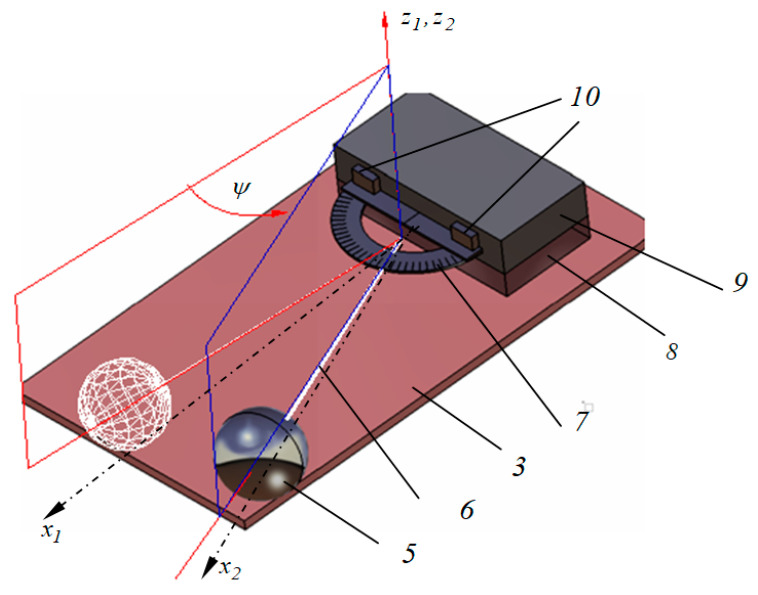
Scheme for measurement of precession angle.

**Figure 10:**
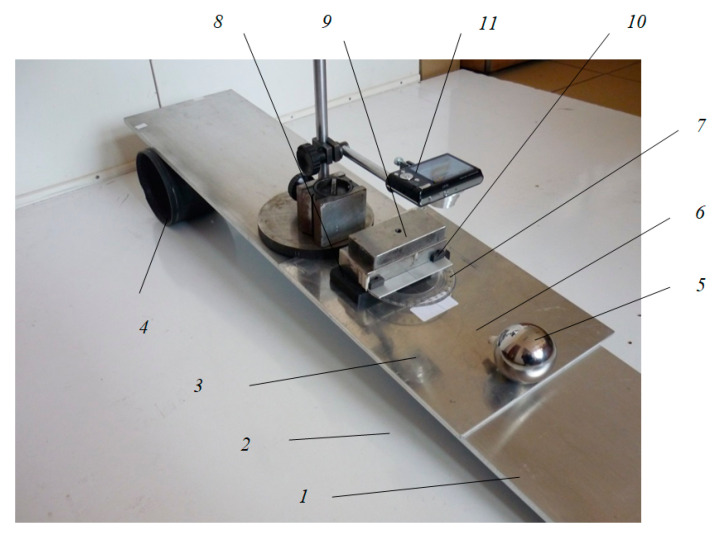
Experimental device.

The principle scheme of the experimental device presented in Figure 1 is detailed in Figure 9. Since the pure rolling condition is fulfilled, between the precession angle *ψ* and the intrinsic rotation angle *φ*, a relation exists (Equation (19)), and the position of the pendulum will be completely described by any of the two parameters. From an experimental point of view, the precession angle is more convenient to be measured (between the fixed axis *x*_1_ and mobile axis *x*_2_) while the intrinsic rotation angle is measured between two mobile axes (*x*_3_ and *x′*). From Figure 9, it is noticed that the axes *x*_1_ and *x*_2_ are normal to the axis *z*_1_ ≡ *z*_2_. The same *ψ* angle is formed by the two planes made by the axis *z*_1_ ≡ *z*_2_ and the axis *x*_1_ and *x*_2_, respectively; see Figure 9. The ball, 5, oscillates on the plate to be tested, 3; a rubber plate, 8, is positioned with the edge to the center of the fulcrum, *O*. A rectangular prismatic steel body, 9, is positioned on top of the rubber plate. A protractor, 7, is attached to the body, 9, by means of two magnets, 10. The magnets permit positioning the protractor, 7, both in the plane parallel to the plate, 3, and on the normal direction to the plate, for a minimum distance from the wire, 6, and for obtaining the actual size of the *ψ* angle. The oscillations of the wire are filmed with a camera, 11. The actual device is shown in Figure 10.

The steps made for an experimental test are as follows:

- The plate to be tested, 3, is positioned on top of the duralumin plate, 1;
- The tilting angle of the device to the imposed angle *α* is obtained by moving the adjusting cylinder, 5 (the cylinder is moved until the short mobile edge of the plate, 1, reaches the height *h* = *l*∙sin*α*);
- The ball, 5, is removed from the equilibrium position by a small *ψ*_0_ angle (less than *15deg*) and let to oscillate freely while rolling over the plate, 3; it is observed that the angular amplitude of the oscillation decreases in time and finding the manner of the angular amplitude damp in time is the goal of the test;
- The displacement of the wire, 6, with respect to the protractor, 7, is filmed using a camera, 11, focused on the region where the distance of wire–protractor is minimum;
- The film is transferred to a computer and the images are analyzed frame by frame to obtain the values of the extreme elongations and the instants when they occur;
- In the first stage, these experimental values for time and amplitude were measured manually by a human operator, using QuickTime software; this step was time-consuming and tedious;
- To overcome this inconvenience, an image analysis code was developed (presented in Section 2.3.2) and thus the position of the wire, the times and the extreme angular elongations could be accurately found.

The last step concerns obtaining the value of the coefficient of rolling friction *s_r_* from the experimental data. The proposed method must be validated by comparison to other results from the literature. Since most of the papers present results for the hypothesis of proportionality between the rolling friction torque and normal force, we accepted the same hypothesis for comparing the results. On the same graph, there were plotted both the experimental data for the variation in time of angular amplitude and the theoretical plot of the equation of motion, corresponding to different values of the theoretical coefficient of rolling friction. The solution that best interpolates the experimental data was kept as the value of the experimental coefficient of rolling friction.

#### 2.3.2. Software for Elongation Measurement Using Automatic Image Processing

Since the methodology for angular amplitude estimation by a human operator requires cumbersome work, it was decided that a computer program that uses image processing techniques should be written to estimate the angular elongations of the conical pendulum. The software processes the video and outputs a file with the values of time t and angular elongations at the t instant. The file format is CSV to be straightforwardly used by spreadsheet applications.

**Figure 11:**
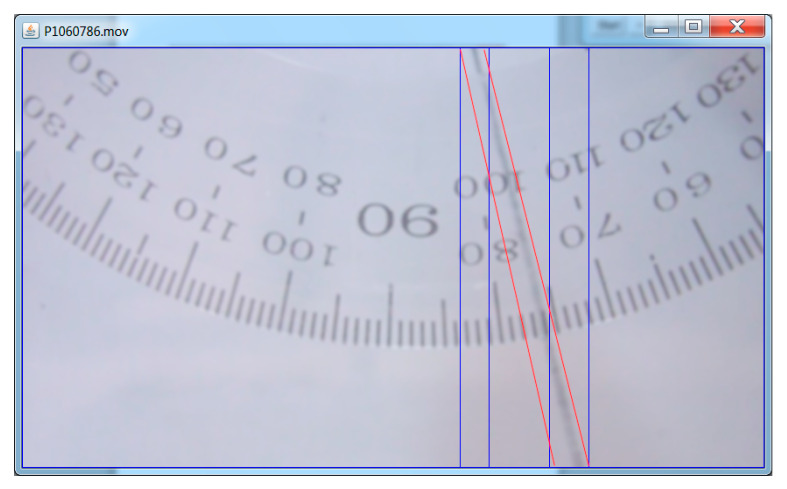
Experimental measurement of the angular amplitude of the pendulum.

The software accomplishes the following processing steps:

Splits the video into constituent frames. For each frame, it performs the following operations:

- detects the moving object, i.e., the wire, partitions it into zones of interest(rectangles) and surrounds the pixels of the wire with a red quadrangle (as shown in Figure 11); due to binarization of image, it is possible that the wire is represented by several disconnected portions, so a number of morphological operations of dilation and erosion are executed;
- in order to establish the orientation of the wire, the coefficients of the regression line passing through the pixels cloud of the wire image in the frame (located in the red polygon on Figure 5) will be determined; these coefficients will be computed based on the coordinates of the wire pixels within the screen (*x_i_*, *y_i_*); thus, having the equation of the regression line,

(35) $$y_{i}=A\;x_{i}+B$$

the angular elongation relative to the screen is determined.

The following information is recorded in the CSV file: the time in seconds (with three decimals) taken from the timestamp of the analyzed frame and the angle in radians relative to the OX axis of the frame.

**Figure 12:**
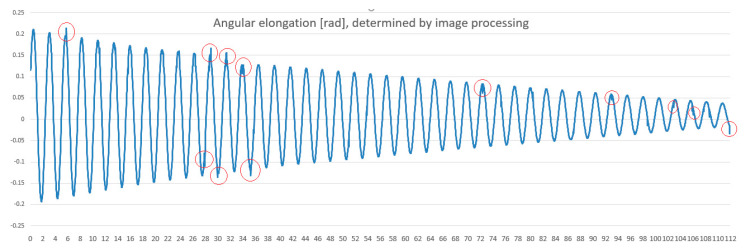
Plot of the angular elongation variation versus time as resulted in the first stage of image processing program elaboration.

Subsequently, all values in the file are scaled considering the relative orientation of the wire in its equilibrium position. The image processing program detects the position of the wire for each frame of the video. To this purpose, the software uses the OpenCV [27] library and the JavaCV [28] interface required to achieve the link between the program written in Java code and the OpenCV library. In Figure 12, there are some values marked with red circles that are erroneous from the estimation point of view. These values prove the limits of the method, which is influenced by the quality of the video.

The following approach was adopted to correct such values:

- the program establishes for each frame the value of the angular elongation after image processing;
- an estimated value is also determined depending on the value from the previous frame; if the difference in absolute value between the established value and the estimated one is greater than a certain threshold, then the automatic processing stops at this frame, which is displayed together with the graphical representation of the two lines (the regression one and the one corresponding to the estimated value);
- the operator examines the frame at which the processing stopped; if there is a discrepancy between the orientation of the wire and the calculated regression line, the operator can manually select two marks by clicking on the image of the wire, and the program calculates the angular elongation for this frame based on the line passing through the two chosen marks and resumes the automatic processing.

This methodology was chosen as it was considered preferable to work with unprocessed data. The filtering algorithm for the calculus of estimated value was used only to decide to stop the video at a frame where there was a noticeable deviation from the accepted threshold. For the completed experiments, the operator’s involvement is reduced to 10–20 frames, mainly where the image of the wire is less clear. These are small in number compared to the thousands of frames which should otherwise be estimated manually. The protractor was initially used for the manual reading, frame by frame, of angular elongation, by a human operator. In the automatic image processing for the detection of the wire, the image of the protractor is not taken into consideration, since the moving object is analyzed and the protractor is immobile. However, the protractor was used to validate the results produced by the computer program. Subsequently, when an acceptable degree of precision of the delivered results was proven, the protractor was ignored. Using the data from the CSV file which contains the values of angular elongations and the corresponding instants, the time moments of maximum elongation and the values of angular amplitudes for each oscillation can be easily identified.

This program that has at the input a video clip with the oscillating motion of the pendulum and whose output is a CSV file containing the angular elongations and various time moments was executed on a laptop with microprocessor Intel (R) Core (TM) i7-8550U CPU @ 1.80GHz, and 8M RAM, running the Windows operating system.

### 2.4. Experimental Results

**Figure 13:**
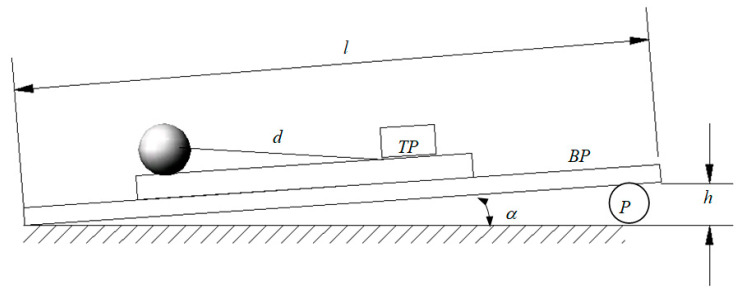
The angle of the inclined plane—truthful valuation.

The materials used for the method are a bearing steel ball (radius *r* = 5/4″ = 0.03175 m) and a plate to be tested, TP. For the first tests, a 6 mm thick glass plate was used. In order to obtain a well-controlled inclination of the plate, a much longer aluminum plate, BP, of length *l* = 1.500 m was used to support it. A cylindrical part, P, was placed beneath the aluminum base and the level difference between the two ends of the plate was *h* = 0.142 m. Consequently, the angle of inclination (Figure 13) is well-controlled and has the following value:

(36) $$\alpha =a\sin\frac{0.142}{1.500}=5.432{}^{\circ},$$

The length of the pendulum is denoted by *d.*

Tests were performed for three different values of the pendulum length *d.* The *d* parameter is difficult to size, so the distance L between the fulcrum and the ball-glass contact point is measured, these two points being reachable. Then, the simple relation is used for finding the *d* length:

(37) $$d=\sqrt{L^{2}+r^{2}}.$$

**Figure 14:**
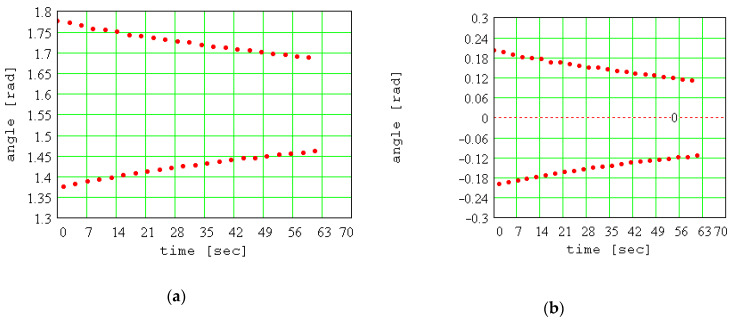
Experimental evidence of the decrease in angular amplitude of the pendulum: (**a**) initial values; (**b**) with respect to the equilibrium position.

The values found for *d* for the three experimental tests are as follows: *d* = 0.140 m; *d* = 0.195 m; *d* = 0.240 m. The experimental values of maximum oscillation amplitudes are represented graphically. In Figure 14a, there are plotted the results for the first test (*d* = 0.140 m). Establishing the equilibrium position of the pendulum is a major issue of the method. To solve it, the following function is created:

(38) $$F(\psi )=\sum_{k=1}^{n}{\left(\psi_{k}-\psi \right)}^{2}.$$

The searched equilibrium position corresponds to the value *ψ_0_* that gives the minimum of the function. The value *ψ_0_* results from the following equation:

(39) $${\frac{dF(\psi )}{d\psi }|}_{\psi =\psi_{0}}=0,$$

where *n* represents the number of experimental points. Now, with known value *ψ*_0_ for the position of equilibrium, the decrease in the angular amplitude of the pendulum can be represented, as in Figure 14b.

**Figure 15:**
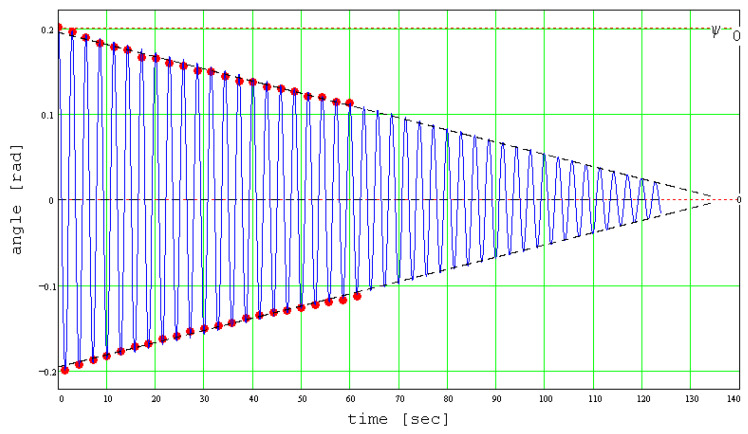
Theoretical angular elongation and values of experimental angular amplitude.

Next, taking *β* = 1, the value of *s_r_* is found imposing the condition that the theoretical curve passes as closely as possible to the experimental points. Figure 15 presents the experimental and theoretical data and their excellent superimposition.

**Figure 16:**
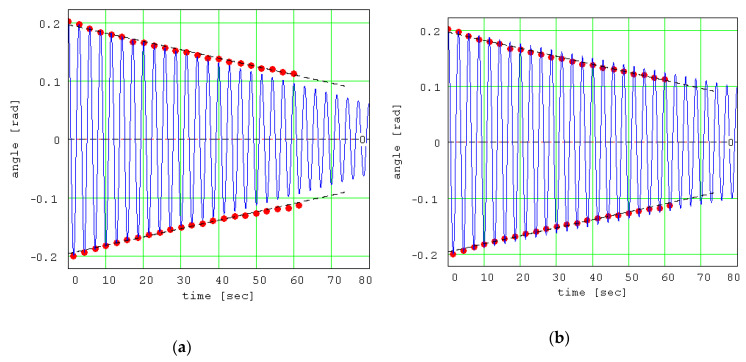
Theoretical signals for different values of the dimensional coefficient of rolling friction: (**a**) Theoretical signal for *s_r_* = 3.7 × 10^−6^ m; (**b**) Theoretical signal for *s_r_* = 2.7 × 10^−6^ m.

From Figure 15, it can be observed that the amplitude of the theoretical signal decreases linearly with time, and this tendency is supported by the experimental data. Dzhiladvari [16] found the same dependency for the final stage of oscillations. On the same plot, there were represented the two regression lines that interpolate the experimental data. These lines are also the envelopes of the theoretical signal. In Figure 15, the theoretical signal is shown for the coefficient of rolling friction *s_r_* = 3.2∙10^−6^ m. It should be mentioned that the method is strongly dependent on the coefficient *s_r_*, as seen in Figure 16, where the values were modified in addition and subtraction with small quantities and the plots (blue) detach from the regression lines (black dash).

**Figure 17:**
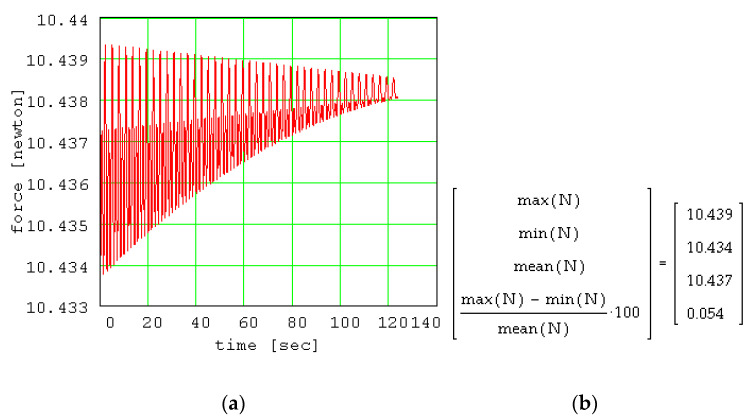
The variation of the normal force: (**a**) plot of normal force variation; (**b**) Mathcad results for extreme and mean values of normal force.

**Figure 18:**
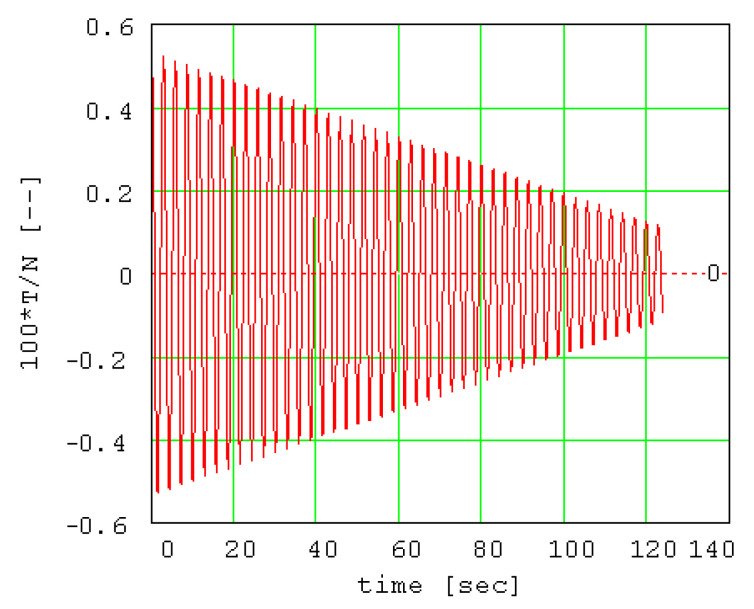
The ratio between friction force and normal force (*T/N*).

The variation of the normal force is presented in Figure 17 and the extreme and mean values are also given. The normal force can be considered approximately constant because it was demonstrated that the maximum variation of the normal force during the oscillation process is 0.054% from the medium value. From Equation (28), it results that the rolling friction torque will have a similar behavior, regardless of the value of the β exponent. The ratio between the friction force and the normal force *T/N* is represented in Figure 18 in order to estimate the validity of the pure rolling condition, expressed by Equation (34). From the plot, it is remarked that the values of the ratio are one hundred times smaller than the values of the coefficient of sliding friction. This is an important feature of the conical pendulum compared to the planar pendula, [17,18] for which, for amplitude values greater than 15°..20°, the sliding friction replaces the rolling friction.

In Table 1, there are presented the values obtained for the coefficients of rolling friction, dimensional *s_r_* [13,17,29,30,31,32,33] and dimensionless *μ_r_* [34,35]:

(40) $$\mu_{r}=s_{r}/r$$

between a steel bearing ball (*r* = 0.03175 m) and different materials: plates made of quenched and tempered steel, glass, aluminum, copper, polycarbonate, carbon fiber composite and glass fiber fabric covered glass plate. The parameter *a* is the modulus of the slope of the straight lines which envelope the theoretical signal.

**Figure 19:**
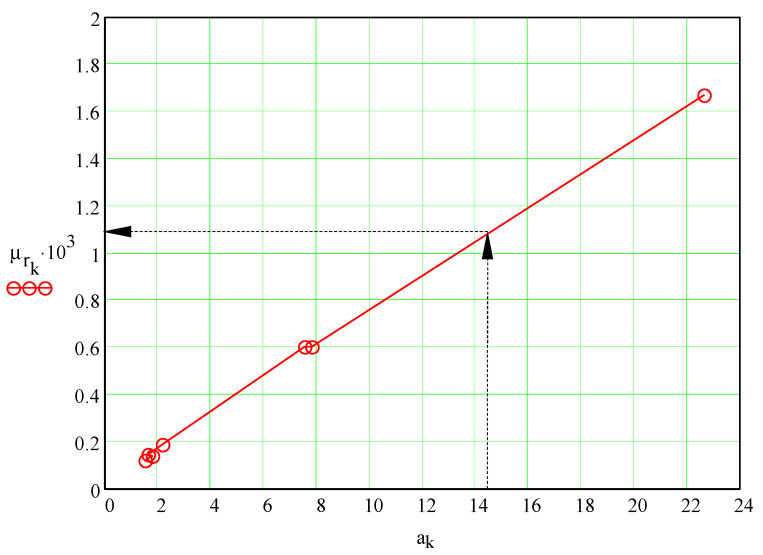
The coefficient of rolling friction versus the slope of the envelope straight line.

Figure 19 presents the dependency of the coefficient of rolling friction *μ_r_* on the modulus of the slope *a* of the straight line enveloping the theoretical signal. The plot shows the proportionality between parameters. This result substantially simplifies the methodology of finding the coefficient of rolling friction: practically, for the same test device geometrical parameters (inclination of the plate, ball radius, pendulum length), two points are sufficient for representing the plot. For a material to be tested, the experimental data obtained from oscillations of the pendulum (the slope of the regression line of angular amplitudes) together with the *μ_r_(a)* plot permit us to obtain the value of the rolling friction coefficient. This direct methodology avoids the guess steps used in integration of the dynamic Equation (33).

## 3. Discussions

The commercial tribometers are dedicated to the estimation of friction and wear, the measurements being based on the pin-on disc scheme, where sliding friction and wear are evaluated [36]. Even for cases where standard methodology and testers exists, there are scientists who prefer to design and build new equipment for tribological tests [37]. Concerning the rolling friction, only for the tire–road pair there are standard tests [38,39]—that is, for compliant materials which exhibit large deformations. For the pairs of materials frequently met in mechanical applications which present important normal contact stiffness (steel, glass, carbon composites, plastics, etc.), there are no standard methods and instruments for determining the parameters characteristic to rolling resistance. Therefore, over time, a series of methods and devices have been proposed by researchers [32,33,34,35,40,41]. Our work joins this trend for finding a simple, rapid and inexpensive method and device for estimating a parameter that characterizes the rolling resistance between two different materials. The equation of motion is deduced for the spatial problem.

The paper presents a method for the estimation of the rolling friction torque, based on the experimental study of the oscillatory motion of a conical pendulum. The conical pendulum is made of a metallic ball attached to an inextensible wire. The ball rests on a flat surface inclined with respect to the horizontal direction. The conical pendulum is obtained when the end of the wire is fixed in a point from the surface. Attaching the end of the wire to the plane causes the spinning moment between the ball and the plane to be zero. When the ball is removed from the equilibrium position and then set free, it oscillates. For small initial amplitudes, the motion of the ball with respect to the plane is pure rolling. The motion of the ball can be entirely determined by the angle between the current position of the wire and the equilibrium position.

For the motion of a rigid body with a fixed point, Euler’s equations must be used. Applying Euler’s equations for the conical pendulum, there are found the positional parameters (precession angle and intrinsic rotation angle) and the expressions of normal and tangential reaction forces acting in the point of contact. The pure rolling condition is expressed as proportionality between the two angles and allows for complete solving of the dynamics of the pendulum. Especially important is the dependency of the reaction forces on the precessional angle, namely the lack of the second derivative of this parameter. (It should be mentioned that for planar pendula, this second derivative occurs in expressions of the reaction forces, as shown in the planar cycloidal pendulum). This is an important advantage because one can accept any dependency between the moment of rolling friction and normal reaction without obtaining intricate expressions from which the second derivative cannot be analytically solved, which is a requirement of the numerical methodology of Runge–Kutta for the integration of the equation of motion. Additionally, from the analysis of the equation of motion, it is noticed that the moment of rolling friction appears explicitly in the expression of the angular acceleration of the pendulum. Thus, the theoretical model becomes a useful theoretical instrument for future research. One can accept any expression for the rolling friction moment as function of velocity, position and time and, finally, the constants from the expression are found from the condition of optimum interpolation of experimental data with the theoretical signal resulting from the theoretical model.

The rolling friction torque was assumed to depend on the normal force obeying a power law function. The hypothesis of proportionality between the rolling friction torque and normal force was accepted as encountered in other works [42,43,44,45] and confirmed by experimental results. In the literature, the dimensionless coefficient of rolling friction is widely met [34,35,40,41,42,43], but it is related to the dimensional one by the radius of the ball.

The motion of the center of the ball is a damped one. A video camera records the motion of the wire with respect to a protractor, and afterward, using software, the moments of the wire’s maximum angular elongation are found. In the first stage, these instances were established by human operators, which was a tedious task. To overcome this drawback, a computer code was written in Java to analyze each frame of the film with the oscillating motion of the conical pendulum. By applying digital image processing techniques, the angular elongations were determined at various times. This approach for measuring the angular elongation considerably reduced the effort of the human operator and made possible the running of more experiments. The accuracy of the measurements was satisfactory for the study. The program detects the few possible errors caused by the poor quality of some frames and prompts the operator’s decision. The experimental data are represented graphically, where it is observed that the decrease in the wire’s angular amplitude is linear, as predicted by the theoretical model.

Several experiments are made to find the values of the coefficient of rolling friction between a steel ball and a plate made of a different material (glass, copper, aluminum, polycarbonate, etc.) and the experimental data are confirmed by a plot of the theoretical elongation of the wire. The theoretical model is especially sensitive to the variation of the coefficient of rolling friction. The validation of the method was performed by testing the steel–steel pair of materials, widely used in the rolling bearing industry. The studies on the subject consider numerous parameters (radii of rolling bodies, elastic characteristics, surface roughness, adhesions, thermal treatment, etc.) and the variety of the values of these parameters makes the comparison possible as qualitative aspect. For instance, the steel–steel pair may present values of 0.002 for untreated steel [35] or 0.0002–0.0004 for thrust bearing parts [32], in good agreement with the results of the present work, 0.000147. Tests for steel–polycarbonate pairs of materials achieved the value of 0.00075 in [33], which is comparable to the value of 0.0006 achieved in the present tests.

An interesting observation was revealed after plotting the dependence between the coefficient of rolling friction and the slope of the enveloping lines of the theoretical signal which interpolates the experimental data. The modulus of the slope of the enveloping straight line is proportional to the coefficient of rolling friction and this can simplify the methodology of finding the coefficient of rolling friction between two materials. In fact, two points from the plot obtained via the initial methodology are sufficient for obtaining the constant of proportionality of the device. Subsequently, for any other material used as plate from the ball–plate pair, from the experimental data, the slope of the regression line concerning the decrease with time in the angular amplitude of the pendulum is enough for calculating the coefficient of rolling friction.

## 4. Conclusions

The coefficient of rolling friction is an important parameter influencing the tribological behavior of mechanical systems. The coefficient of rolling friction is a consequence of the deformations of the bodies in the contact region. The majority of the studies concerning the rolling friction discuss the materials used for vehicle tires. For these materials, the deformations for nominal forces are large and comparable to the dimensions of the contacting bodies, and finding the deformations is not a difficult task. For other materials used in engineering (steel, iron, metallic materials, glass, a range of composites, etc.) which are less compliant, finding the coefficient of rolling friction is a difficult objective. Actually, due to the complexity of the subject, there is not yet a parameter or methodology widely recognized for the estimation of this parameter. The present work proposes a simple, inexpensive, quick method for experimental measurement of the coefficient of rolling friction between a steel ball and a plate, made of similar or dissimilar materials.

Two main stages must be carried out for obtaining the coefficient of rolling friction. In the first step, a conical pendulum is constructed using the ball and the inclined plate and it is observed that the oscillations are damped. The experiments show that the damping with time of the oscillations amplitudes is a linear one. This operation was initially carried out by a human operator by direct observation of the motion of the pendulum’s wire with respect to a protractor. A computer code was written in Java to analyze each frame of the film with the oscillating motion of the conical pendulum. The program detects the few possible errors caused by the poor quality of some frames and prompts the operator’s decision.

In the second stage, the theoretical law of oscillation of the pendulum is found using a theoretical model, depending on the coefficient of rolling friction. From the condition that the experimental data and the theoretical signal must superpose, the actual value of the coefficient of rolling friction is found.

In order to validate the method, the hypothesis of the model is that the moment of rolling friction is proportional to the normal force from the contact. Thus, the results from our work are in agreement with the values presented in other papers in the literature. It is confirmed that for less compliant bodies, the coefficient of rolling friction can be even 10,000 times smaller than the characteristic dimensions of the bodies and the difficulty of estimating this parameter is evidenced.

## Figures and Tables

**Table 1:** Values of rolling friction characteristics for a steel ball rolling over different materials.

Material of the Plate	$s_{r}\cdot 10^{6}$ (m)	$\mu_{r}\cdot 10^{3}$ (-)	$a\cdot 10^{3}$ (rad/s)
**Steel**	4.508	0.142	1.656
**Glass**	3.714	0.117	1.556
**Aluminum**	4.445	0.140	1.811
**Copper**	18.986	0.598	7.587
**Polycarbonate**	18.986	0.598	7.823
**Carbon fiber composite**	5.810	0.183	2.189
**Glass fiber fabric over glass plate**	53.023	1.670	22.65

**Figure A1:**
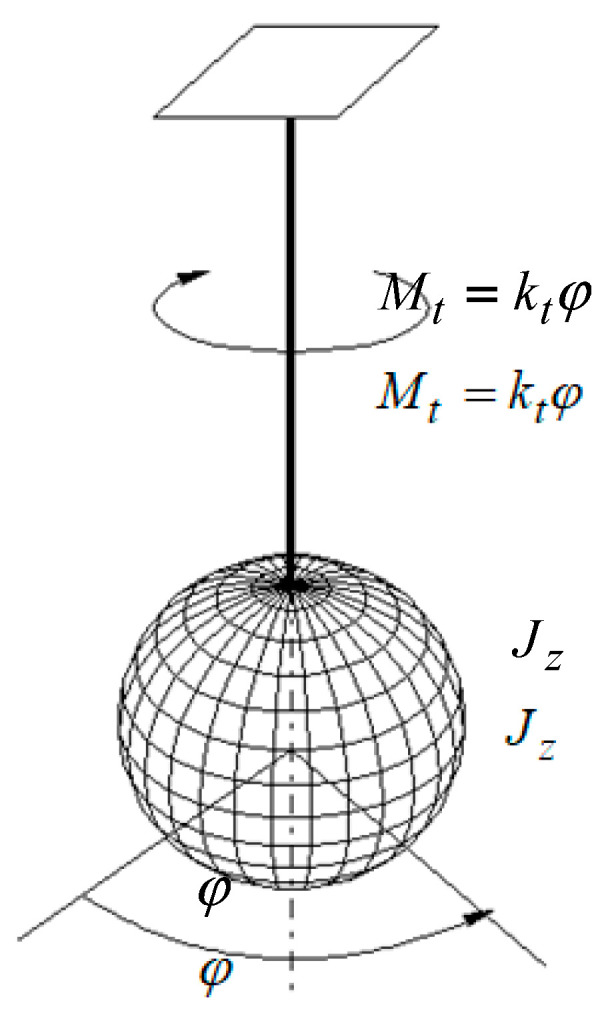
Torsional pendulum.

**Figure A2:**
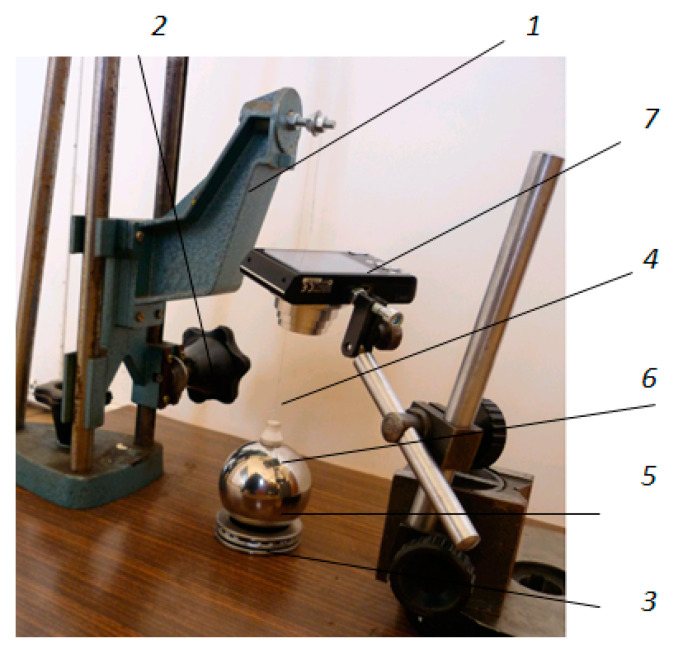
Set-up for torsional oscillations.

## Appendix A

In order to estimate the rigidity of the wire, the torsion pendulum method is applied. The torsion pendulum is made with the same ball and the same wire used for the conical pendulum; see Figure A1. By denoting the torsional rigidity of the wire *k_t_*, when the pendulum rotates with a *φ* angle with respect to the equilibrium position, the wire is subjected to the moment of torsion *M_t_ = k_t_φ* that opposes the torsion of the wire.

The equation of motion of the pendulum is

(A1) $$J_{z}\ddot{\varphi }=-M_{t},$$

or

(A2) $$J_{z}\ddot{\varphi }+k_{t}\varphi =0.$$

The equation is written under the following form:

(A3) $$\ddot{\varphi }+\frac{k_{t}}{J_{z}}\varphi =0.$$

The torsional oscillation pulsation is ω and is given by the following ratio:

(A4) $$\frac{k_{t}}{J_{z}}=\omega^{2}=\frac{4\pi^{2}}{T^{2}},$$

where T is the torsional oscillation period. It results in the following:

(A5) $$k_{t}=\frac{4\pi^{2}}{T^{2}}J_{z}\mu_{r}=s_{r}/r.$$

The moment of inertia of the ball *J_z_* is known (Equation (A4)) and for finding the *k_t_* constant, the period T must be obtained by experimental means. The device is presented in Figure A2: the end of the wire is attached to the mobile carrier, 1, which moves vertically and is fixed by the rosette, 2. A thrust bearing, 3, is placed on the board of the device, coaxial to the wire, 4. After the ball, 5, takes the rest position, it is slowly lowered till it contacts the upper ring of the bearing. Next, the upper ring of the bearing is rotated a certain number of rotations. The rotation motion is filmed with a camera, 7. The carrier is gradually raised until the ball detaches from the ring. The ball starts to rotate about the wire and the mark, 6, glued to the superior part of the ball helps in identifying the rotations. The film is used to find the value of the torsional oscillation period.

For *J_z_* = 4.217 × 10^−4^ kg∙m^2^ and a wire length of 0.2 m, the experimental torsional oscillation period is *T* = 205.3 s and the resulting value of rigidity constant is *k_t_* = 3.95 × 10^−7^ N∙m/rad. For an initial precession amplitude *ψ*_0_ = 15 deg, an amplitude of self-rotation *φ*_0_ = 94.5 deg = 1.65 rad corresponds. This maximum angle of torsion of the wire gives the moment of torsion *M_tmax_* = *k_t_φ*_0_ = 6.51 × 10^−7^ N∙m. For the conical pendulum, the smallest value of rolling friction was obtained for the steel–glass pair, *s_r_* = 4.5 × 10^−6^ m, and the corresponding rolling friction torque calculated according to the proposed model is *M_r_* = *s_r_mg* = 4.61 × 10^−5^ N∙m. Calculating the ratio (*M_tmxa_/M_r_*)∙100 = 1.41%, it can be concluded that the effect of the moment of torsion of the wire is negligible.
